# Modulation of adipose tissue metabolism by microbial-derived metabolites

**DOI:** 10.3389/fmicb.2022.1031498

**Published:** 2022-12-09

**Authors:** Wenyun Liu, Ge Yang, Pinyi Liu, Xin Jiang, Ying Xin

**Affiliations:** ^1^Jilin Provincial Key Laboratory of Radiation Oncology & Therapy, The First Hospital of Jilin University, and Key Laboratory of Pathobiology, Ministry of Education, Jilin University, Changchun, China; ^2^Key Laboratory of Pathobiology, Ministry of Education, Jilin University, Changchun, China; ^3^Jilin Provincial Key Laboratory of Radiation Oncology and Therapy, The First Hospital of Jilin University, Changchun, China; ^4^NHC Key Laboratory of Radiobiology, School of Public Health, Jilin University, Changchun, China

**Keywords:** gut microbiota, obesity, adipose tissue, short-chain fatty acids, bile acids, tryptophan, trimethylamine-N-oxide, lipopolysaccharides

## Abstract

Obesity and its complications, including type 2 diabetes, cardiovascular disease, and certain cancers, have posed a significant burden on health and healthcare systems over the years due to their high prevalence and incidence. Gut microbial derivatives are necessary for the regulation of energy metabolism and host immunity, as well as for maintaining homeostasis of the intestinal environment. Gut flora metabolites may be a link between gut microbes and diseases, such as obesity, and help understand why alterations in the microbiota can influence the pathophysiology of human disease. This is supported by emerging evidence that microbial-derived metabolites, such as short-chain fatty acids, bile acids, tryptophan, trimethylamine-N-oxide, and lipopolysaccharides, can be beneficial or detrimental to the host by affecting organs outside the gut, including adipose tissue. Adipose tissue is the largest lipid storage organ in the body and an essential endocrine organ that plays an indispensable role in the regulation of lipid storage, metabolism, and energy balance. Adipose tissue metabolism includes adipocyte metabolism (lipogenesis and lipolysis), thermogenesis, and adipose tissue metabolic maladaptation. Adipose tissue dysfunction causes the development of metabolic diseases, such as obesity. Here, we review the current understanding of how these microbial metabolites are produced and discuss both established mechanisms and the most recent effects of microbial products on host adipose tissue metabolism. We aimed to identify novel therapeutic targets or strategies for the prevention and treatment of obesity and its complications.

## Introduction

Over the past 40 years, obesity has become an urgent public health issue because it is a major contributor to several diseases, including cardiovascular disease (CVD), type 2 diabetes mellitus (T2DM), non-alcoholic fatty liver disease (NAFLD), and several types of cancers (Lauby-Secretan et al., 2016; Dwivedi et al., 2020; Pagidipati et al., 2020; Petrelli et al., 2021). According to “the Dietary Guidelines for Chinese Residents,” more than half of all Chinese adults will be overweight or obese by 2022. According to the World Health Organization (WHO), obesity has reached an epidemic level in Europe. Therefore, it is crucial to find innovative ways to treat obesity and its associated complications.

Recent research has focused on the gut microbiota and its metabolites. The gut flora comprises various microbial communities. Furthermore, the gut microbiota contributes greatly to the host metabolism, immune system, and brain activity (O’Hara and Shanahan, 2006; Zhang and Davies, 2016). Gut microbes exert their effects through direct cell-to-cell interactions and metabolites produced by microbes or transformed from environmental or host molecules (Agus et al., 2018). Recent studies have revealed the relevance of gut microbiota-derived metabolites in metabolic diseases and their effects on adipose tissue, muscle, and liver metabolism (Wikoff et al., 2009; Chen et al., 2019; Virtue et al., 2019). There are two main categories of gut microbiota-derived metabolites, depending on the type of substrate utilized by the intestinal flora. One is from carbohydrates (including short-chain fatty acids and ethanol), and the other is from protein fermentation (including ammonia, indoles, hydrogen sulfide, and branched-chain fatty acids; Canfora et al., 2019). Some other metabolites, such as secondary bile acids, lipopolysaccharides, dimethylamine, and trimethylamine, have also raised great concern. Adipose tissue, a metabolic regulator of energy homeostasis, serves to store energy (Chait and den Hartigh, 2020). Here, we selected five microbial products highly relevant to adipose tissue metabolism: short-chain fatty acids (SCFA), bile acids, tryptophan metabolites, trimethylamine-N-oxide (TMAO), and lipopolysaccharides (LPS). In this review, we summarize their roles in regulating adipogenesis, lipolysis, adipose tissue thermogenesis, and inflammation (Table 1) and aim to provide new research threads in the field of gut microbiota associated with human metabolic diseases.

**Table 1:** Microbial-derived metabolites modulate adipose tissue metabolism.

Metabolites	Cell type/tissue type/subject	Receptors and pathway	Outcome	Refs
SCFAs (acetate, propionic, and butyrate)	3 T3-L1; Porcine vascular smooth muscle cells; Male C57BL/6 J mice	GPR43	↑Adipogenesis; ↑PPAR-γ2, LPL, FABP4, SREBP-1c, C/EBPα/β, GLUT4, LPL, PPAR-γ, GPAT4, DGAT1, and DGAT2 mRNA	Toscani et al. (1990), Hong et al. (2005), Aguilar et al. (2018), and Huo et al. (2022)
SCFAs (acetate, propionate, butyrate, and valerate)	hMADS	GPR43	↓Lipolysis; reduce intracellular lipid spillover; ↓HSL	Brown et al. (2003), Ohira et al. (2013), Byrne et al. (2015), and Chait and den Hartigh (2020)
Acetate	SAT	GPR43; G(i/o)βγ/PLC /PK C/MAPK	↑Browning adipogenesis; ↑aP2, PPAR-γ, PRDM16, UCP1, and DIO2 mRNA	Hu et al. (2016) and Sahuri-Arisoylu et al. (2016)
Bile acids (TCA and DCA)	C57BL/6 J mice	TGR5; cAMP-PKA-CREB	↑WAT thermogenesis; ↑UCP1, CKMT2	Watanabe et al. (2006) and Portincasa et al. (2020)
KYNA	C57BL/6 J mice	GPR35	↑Cellular respiration; ↑β-adrenergic signaling; ↑Thermogenic genes in adipose tissue	Pyun et al. (2021)
5-HT	C57BL/6 J mice	HTR2B	↑Lipolysis; ↑Phosphorylation and activity of hormone-sensitive lipases	Voigt and Fink (2015)
5-HT	3 T3-L1; C57BL/6 J mice	HTR3	↓Thermogenesis of BAT	Sumara et al. (2012)
5-HT	3 T3-L1; Rat adipose tissue	HTR2A	↑Lipogenesis; ↑FASN	Sumara et al. (2012) and Oh et al. (2015)
TMAO	Adult female mice	FMO3	↑WAT browning	Petriello et al. (2016)
LPS	Ob/ob mice	CB1	↑WAT browning	Alexander et al. (2019)

SCFAs, short-chain fatty acids; PPARγ2, peroxisome proliferator-activated receptor-γ; LPL, lipoprotein lipase; FABP4, fatty-acid-binding protein; SREBP-1c, sterol regulatory element-binding protein 1C; C/EBPα/β, CCAAT-enhancer-binding proteins α/β; GLUT4, glucose Transporter 4; GPAT4, glyceryl transferase 4; DGAT1/2, diacylglycerol O-acyltransferase homolog 1/2; hMADS, human multipotent adipose tissue-derived stem cells; HSL, hormone-sensitive lipase; SAT, subcutaneous adipose tissue; G(i/o)βγ, Gi/o-derived free Gβγ subunits; PLC, Phospholipase C; PKC, protein kinase C; MAPK, mitogen-activated protein kinase; aP2, adipocyte fatty-acid-binding protein; PRDM16, positive regulatory domain containing16; UCP1, uncoupling protein 1; DIO2, iodothyrinine deiodinase 2; TCA, taurine-conjugated bile acid; DCA, deoxycholic acid; TGR5, Takeda G protein-coupled receptor 5; WAT, white adipose tissue; CKMT2, Recombinant Creatine Kinase, Mitochondrial 2; HTR2A/2B/3, 5-hydroxytryptamine (serotonin) receptor 2A/2B/3; BAT, brown adipose tissue; FASN, Fatty acid synthase; FMO3, flavin monooxygenase 3; CB1, cannabinoid receptor 1.

## Short-chain fatty acids

### Sources and composition of short-chain fatty acids

Short-chain fatty acids (SCFAs) are largely derived from dietary fiber (Wolfe and Dutton, 2015), which is fermented by gut microbes in the cecum and colon to produce SCFAs (Cummings et al., 1987). The relative amount of each SCFA in the intestine depends not only on the food and type of intestinal flora but also on the transit time through the bowel (Turnbaugh et al., 2009; Wu et al., 2011). The most abundant SCFAs in the human body are produced by different types of fermenting bacteria and include acetate, propionate, and butyrate. Acetate is mainly produced by *Bacteroides*, *Bifidobacterium, Streptococcus, Streptococcus peptica, Clostridium,* and *Rumex coccus*. Butyrate is produced by *Bacteroides, Eubacterium*, and *Clostridium* (Walker et al., 2005; Lu et al., 2016; Nishitsuji et al., 2017). Propionate is produced by *Clostridium* and *Bacteroides* (Derrien et al., 2004; Frost et al., 2014). They also differ in concentration in the human body, with a ratio of approximately 3:1:1 of acetate, propionate, and butyrate in the colon (Binder, 2010). Acetate usually accounts for 60%–75% of the total SCFAs in feces, being the most abundant SCFA in humans (Parada Venegas et al., 2019).

### Receptors for short-chain fatty acids

There are two currently known specific receptors for SCFA: G protein-coupled receptor GPR41 (also known as free fatty acid receptor 3, FFAR3) and GPR43 (FFAR2; Brown et al., 2003). SCFAs are signal transduction molecules for GPR109A and OLFR78 (olfactory receptor 78; Macia et al., 2015; Li et al., 2020). Here, we focus on the classical receptors, GPR41 and GPR43. GPR41 is highly expressed in the human adipose tissue, pancreas, spleen, enterocytes, and enteroendocrine cells. In rodents, GPR41 is primarily expressed in the kidney, colon, spleen, and adipose tissue. GPR43 is expressed in human adipocytes, the liver, heart, pancreatic islets, spleen, and L cells of the large intestine. In addition, GPR43 is expressed in the colonic epithelium and mucosa but not in the colonic muscle and submucosal regions. It is commonly expressed in the adipose tissue, stomach, colon, spleen, and immune cells of mice, which contain white adipose tissue and adipocyte lineage 3 T3-L1 (Mishra et al., 2020). However, its expression in brown adipose tissue remains unclear (Hu et al., 2016, 2018). There is some disagreement in the literature regarding whether GPR41 is expressed in adipose tissues (Zaibi et al., 2010). SCFAs have different receptor affinities in various animal models. In humans, acetate exhibits GPR43 selectivity, whereas propionate and butyrate are nonselective (Hudson et al., 2012). In contrast, SCFAs can activate both GPR41 and GPR43 in mice. Hence, GPR41 and GPR43 may play active roles in adipocyte metabolism (Hudson et al., 2012). GPR43 is coupled to Gi/o and Gq/11 proteins (Le Poul et al., 2003; Stoddart et al., 2008; Mishra et al., 2020). First, activation of the Gi/o protein inhibits adenylate cyclase (AC). AC reduces intracellular cAMP levels but simultaneously activates extracellular regulated protein kinases 1/2 (ERK1/2) of the mitogen-activated protein kinase (MAPK) pathway. ERK1/2 activates the lipogenic transcription factor CCAAAT/CEBPβ. Second, through activation of the Gq/11 protein by increasing levels of GPR43, intracellular calcium ion levels are elevated, and ERK1/2 and other MAPK pathways are activated downstream. Third, activation of β-arrestin2 by GPR43 inhibits the nuclear translocation of the pro-inflammatory transcription factor nuclear factor-κB (NF-κB) and its activation, which reduces the synthesis of pro-inflammatory cytokines, such as IL-1β and IL-6 (Lymperopoulos et al., 2022). GPR41 is easily activated by the longest-chain SCFAs, such as pentanoate with five carbon atoms and hexanoate with six carbon atoms (Le Poul et al., 2003). In contrast to GPR43, GPR41 signaling seems to proceed only through Gi/o proteins. GPR41 promotes ERK1/2 phosphorylation and activation by free Gβγ subunits of Gi/o origin. Additionally, stimulation of GPR41 by SCFAs inhibits AC, which reduces intracellular cAMP synthesis *via* Gai subunit activation (Vögler et al., 2008; Bolognini et al., 2016). Notably, although it is not known whether GPR41 is coupled to the Gq/11 protein, FFAR3 also induces a phosphatidylinositol hydrolysis cascade, stimulates intracellular Ca2+ signaling, and activates PLCβ2/3 *via* Gi/o-derived free Gβγ subunits (Kimura et al., 2011).

### The effects of short-chain fatty acids on adipose tissue metabolism

Available studies have identified the beneficial effects of SCFAs on the body, and supplementation of SCFAs in the diet significantly attenuates weight gain induced by a high-fat diet. SCFAs can promote energy expenditure by increasing lipid oxidation and stimulating gut-derived hormones to regulate glucose homeostasis (Dass et al., 2007; Lin et al., 2012; Tolhurst et al., 2012; Chen et al., 2017; Christiansen et al., 2018). In addition, SCFAs can regulate energy balance through the gut-brain axis (Gabanyi et al., 2022). Thus, SCFAs play a key role in host energy metabolism. Adipose tissues may be an attractive target for microbial SCFAs. Previous research has shown that SCFAs can prevent fat spillage and ectopic fat storage by inhibiting lipolysis and promoting adipogenesis, thereby enhancing the fat storage capacity of adipose tissues (May and den Hartigh, 2021).

Adipogenesis is the process by which adipose precursor cells differentiate into pre-adipocytes, accumulate nutrients, and then differentiate into mature adipocytes; adipocyte proliferation is based on this process. Previous studies have shown that all three major SCFAs can promote lipogenic differentiation of 3 T3-L1 pre-adipocytes. Acetate treatment of 3 T3-L1 pre-adipocytes leads to an increase in the expression of genes related to lipogenic differentiation (Huo et al., 2022). Hong et al. showed upregulation of peroxisome proliferator-activated receptor-γ (PPAR-γ) and GPR43 mRNA expression in 3 T3-L1 cells treated with acetate or propionic acid for 7 days; this effect was counteracted in the absence of GPR43 (Hong et al., 2005). These data suggest that acetate and propionate stimulate adipogenesis *via* GPR43. A moderate reduction in weight gain in obese mice fed a sodium-butyrate-supplemented diet was associated with reduced adipocyte expansion, induced adipogenesis, and lipocalin production. These results were associated with the increased expression of PPAR-γ and the downregulation of NF-κB (Aguilar et al., 2018). The adipogenesis-promoting effect of butyric acid is reflected by an increase in the number of intracellular lipid droplets and the expression of adipogenesis-related genes, such as lipoprotein lipase (LPL) and fatty-acid-binding protein (FABP4; Toscani et al., 1990). In porcine vascular smooth muscle cells, butyrate treatment promoted adipogenesis and lipid accumulation. This was possibly mediated through upregulation of glucose uptake and induction of SREBP-1c, C/EBPα/β, GLUT4, LPL, PPAR-γ, GPAT4, DGAT1, and DGAT2 expression, partially *via* inhibition of HDAC activity (Yan and Ajuwon, 2015). mTORC1 phosphorylation signaling activates SREBP1 activity in adipose tissue and thus affects adipogenesis. Treatment of animals with *Lactobacillus johnsonii* N6.2 and blueberries extract reduced SREBP1 levels. The use of direct mTOR inhibitors to reduce adipogenesis may adversely affect homeostasis *in vivo*; therefore, the use of probiotics and phenolics may be an effective alternative to reduce potential risk factors associated with the metabolic syndrome (Teixeira et al., 2021).

Lipolysis is the sequential hydrolysis of triglycerides by three ester chains, which, in turn, produces glycerol and free fatty acids. SCFAs play an important role in the lipolytic pathway of white adipose tissue. Physiological concentrations of acetate (1 μmol/L–1 mol/L) are responsible for the antilipolytic effect by weakening hormone-sensitive lipase (HSL) phosphorylation in human multipotent adipose tissue-derived stem cells (hMADS) *via* Gi-coupling (Chait and den Hartigh, 2020). *In vivo* studies have also indicated that acetate inhibits systemic lipolysis under pathological conditions. Acute intravenous administration of acetate reduces plasma free fatty acid levels in hyperinsulinemic and obese individuals. Moreover, rectal injection of a mixture of SCFAs reduces free glycerol concentrations and decreases lipolysis in obese individuals (Ohira et al., 2013). To further clarify the mechanism by which SCFAs inhibit lipolysis, Brown et al. screened a conventional ligand library in yeast, identified acetate as an agonist of human GPR43, and confirmed that other SCFAs, such as formate, propionate, butyrate, and valerate, also have this activity in mammalian cells. SCFAs-induced activation of GPR43 may be mediated by reducing intracellular lipid spillover to regulate adipose tissue lipolysis (Brown et al., 2003; Byrne et al., 2015). Butyric acid may function as an HDAC inhibitor of lipolysis (Rumberger et al., 2014). Better controlled studies in humans are needed in the future to reveal the role of SCFAs in energy metabolism.

SCFAs are also involved in the browning of white adipose tissue. Brown adipose tissue (BAT) is found primarily in newborns and anatomically within the interphalangeal, perirenal, and axillary fat depots. BAT is necessary for the production of non-shivering calories (Moreno-Navarrete and Fernandez-Real, 2019), and it increases whole-body energy expenditure by 40%–80% when it is fully active in the body. As a result of increased thermogenic fat activity, insulin sensitivity is greater in mice, and it prevents weight gain and improves metabolic dysfunction. Extracellular vesicles secreted by BAT have also been shown in recent studies to have a protective effect on the exercising heart (Zhao et al., 2022). In the last decade, browning, the process of transforming white adipocytes into brown-like adipocytes, has been considered a potential treatment for obesity (Moreno-Navarrete and Fernandez-Real, 2019; Alipoor et al., 2020). Acetate upregulates the expression of adipocyte protein 2 (aP2), peroxisome proliferator-activated receptor-γ coactivator-1α(PGC-1α), and uncoupling protein-1 (UCP1). Acetate also affects the morphological changes of brown adipocytes during adipogenesis by activating GPR43 and may work on the Gi/o-derived free Gβγ subunits/phospholipase C/protein kinase C/MAPK kinase signaling pathway (Hu et al., 2016). Limited evidence of this was also confirmed by Lu et al., where supplementation of diets with SCFAs resulted in increased GPR43 and GPR41 expression in adipose tissue and decreased expression in the colon (Lu et al., 2016). In mice, treatment of WAT with nanoparticle-delivered acetate inhibited obesity by inhibiting lipolysis and inducing browning (Sahuri-Arisoylu et al., 2016). Furthermore, in a study of obese patients, it was shown that in subcutaneous adipose tissue (SAT), the relative abundance of Firmicutes was positively correlated with brown adipocyte markers (PRDM16, UCP1, and DIO2). In addition, Firmicutes RA was positively correlated with plasma acetate levels. This demonstrates that the gut microbiota composition may be associated with adipose tissue browning in morbidly obese subjects *via* circulating acetate (Moreno-Navarrete et al., 2018). Acetate increases brown fat activity and induces the formation of brown adipocytes. Butyric acid regulates thermogenesis in BAT and subcutaneous white adipose tissue (scWAT) through activation of LSD1 (Wang et al., 2020). There are also some controversial reports on the effect of short-chain fatty acids upon adipose metabolism. One study demonstrated that accumulation of propionic acid promotes obesity and type 2 diabetes by triggering adipocyte autophagy and that acyl coenzyme A synthase short-chain family member 3 (ACSS3) on the inner mitochondrial membrane of brown adipocytes is a key enzyme for propionic acid metabolism (Jia et al., 2022). Also, it has been claimed that reducing acetate in brown adipose tissue at the physiological level directly inhibits brown fat thermogenesis (Sun et al., 2021). Therefore, further studies are needed if SCFA is to be used as a new research target for the browning of adipose tissue.

In the modern medical view, obesity is a chronic systemic inflammation leading to physiological dysfunction of adipose tissue. SCFAs can ameliorate adipose tissue inflammation. Treatment of adipose tissue with propionic acid significantly lowered the levels of inflammatory cytokines and chemokines (IL-4, IL-10, TNF-α, CCL5, and IP-10) in obese individuals. Macrophage markers (such as CD163 and MMP-9) were also significantly downregulated. Further studies revealed that the anti-inflammatory effect of propionic acid on SAT is partially mediated by Gi/o protein-coupled receptors (Al-Lahham et al., 2012; Louis et al., 2014; Canfora et al., 2017). Another *in vitro* experiment showed that SCFAs markedly reduced the expression of TNF-α and IL-6 in monocytes and macrophages from obese subjects while altering the mRNA expression of FFAR and HDAC (Moreno-Navarrete et al., 2018). Butyric acid also appears to exert anti-inflammatory effects. Mice fed an obesity diet (HFD) containing 2.5% butyric acid for 38 weeks showed reduced adipose tissue inflammation compared to HFD controls (Gart et al., 2021). In mice, supplementation with live J115 (a novel butyric acid-producing bacterium isolated from the human gut), but not with pasteurized bacteria, partially counteracted diet-induced obesity development, fat mass gain, insulin resistance, white adipose tissue hypertrophy, and inflammation (Le Roy et al., 2022). Butyrate protected adipose tissue from infiltration by leukocytes in high-fat-fed mice; reduced the expression of IL-1β, monocyte chemotactic protein 1(MCP-1), IL-6, and TNF-α; and restored the production of the insulin-sensitized adipokine lipocalin (Meijer et al., 2010; Chambers et al., 2015). Mechanistically, it may inactivate the MAPK and NF-κB pathways in macrophages and inhibit adipose triglyceride lipase and histone deacetylase (Shimizu et al., 2018). Butyrate and propionate strongly stimulate PPAR-γ activity in intestinal epithelial cells (Kimura et al., 2014), but whether this mechanism exists in adipose tissue is unclear.

SCFAs also regulate leptin production. Leptin is an adipose-derived hormone that regulates a variety of physiological processes, including metabolism, sympathetic nerve activity, reproduction, and immune responses. *In vivo* experiments showed that oral administration of propionate in the physiological range increased circulating leptin levels in mice through GPR41 activation. *In vitro* analysis showed that propionic acid significantly stimulated leptin mRNA expression and secretion in the omentum and subcutaneous adipose tissue. Zaibi et al. showed that acetate and propionate stimulated leptin secretion from mesenteric adipocytes through GPR43 activation (Zaibi et al., 2010). In conclusion, these findings support the possibility that GPR41 and GPR43 regulate leptin production in WAT stimulated by SCFAs. Differences in the mode of administration, target species and metabolic phenotype provide clues to the experimental design and use of SCFAs in adipose management.

## Bile acids

### Signaling pathways in the regulation of bile acid synthesis by intestinal microorganisms

**Figure 1:**
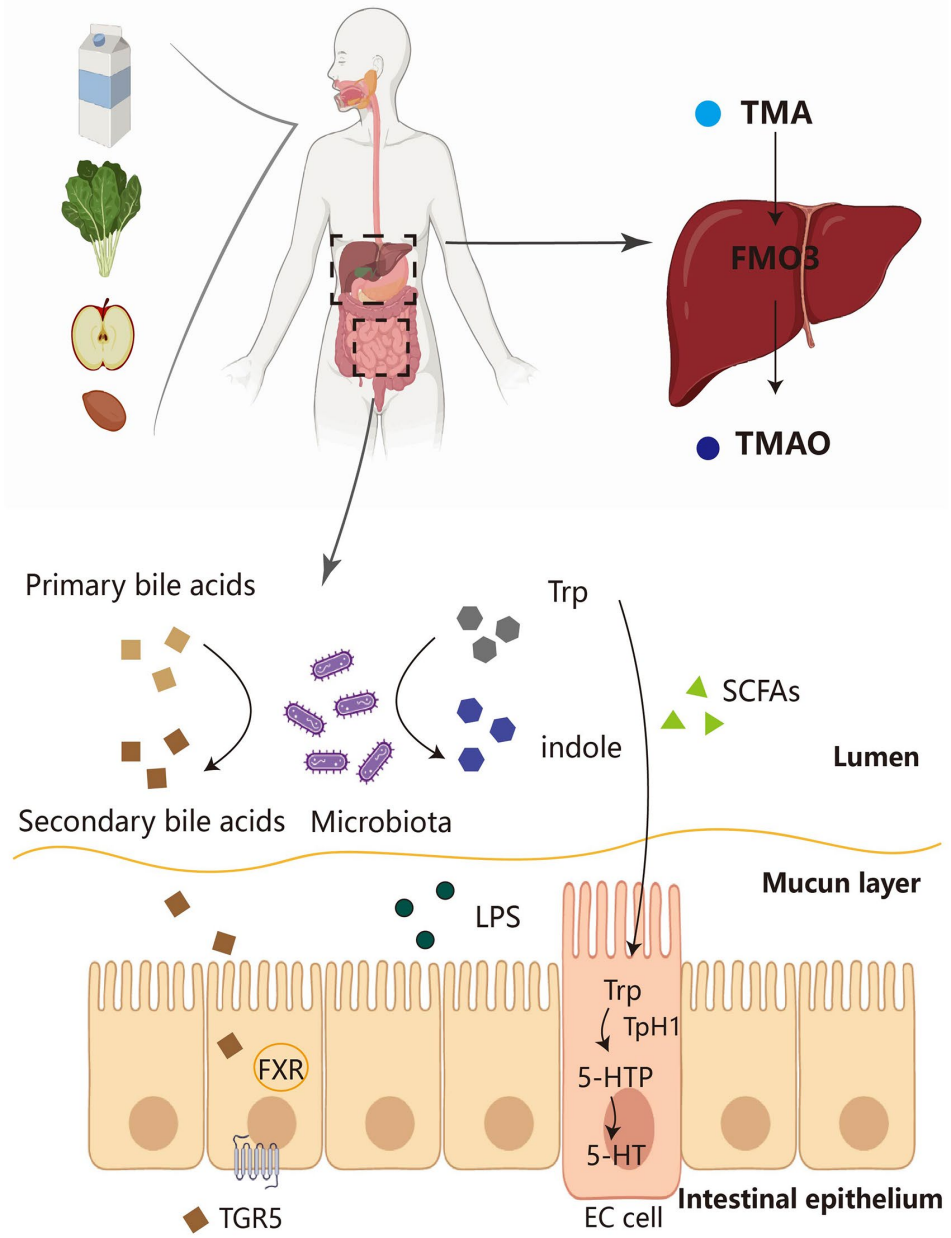
The source pathways of several major gut microbial metabolites. Dietary fiber and protein in food are fermented by the microbiota in the intestine to produce a number of derivatives, including SCFA, Trp and TMA. Primary bile acids can also be converted to secondary bile acids by the intestinal flora, which, in turn, act on FXR-TGR5 for metabolic effects. On the one hand, Trp is converted to indole by microorganisms, and on the other hand, it can be converted to 5-HT by EC cells. TMA is transformed into TMAO by FMO3 in the liver and then acts in adipose tissue. TMA, trimethylamine; FMO3, flavin monooxygenase 3; TMAO, trimethylamine-N-oxide; SCFAs, short-chain fatty acids; LPS, lipopolysaccharides; FXR, farnesoid X receptor; TGR5, Takeda G-protein receptor 5; TPH1, tryptophan hydroxylase 1; 5-HTP, 5-hydroxytryptophan; 5-HT, 5-hydroxytryptamine; EC cell, enterochromaffin cell.

Bile acids (BA) are converted from cholesterol in the liver and play an important role in adipose tissue metabolism. Primary bile acids—such as cholic acid (CA) and chenodeoxycholic acid (CDCA)—are converted into secondary bile acids by bacterial decomposition in the intestine (Figure 1). The secondary bile acids mainly include deoxycholic acid (DCA), lithocholic acid (LCA), and ursodeoxycholic acid (UDCA; Brandl et al., 2017; Pathak et al., 2018). Most of the secreted primary bile acids exist in the enterohepatic circulation, but another portion is not reabsorbed and forms secondary bile acids (Devlin and Fischbach, 2015). In the intestine, the gut microbiota has been shown to alter the composition of BA and the ratio of 12-OH BA to non-12-OH BA, including UDCA, CDCA, and LCA. The intestinal microbiota could convert conjugated BA to the unconjugated form by the action of bile salt hydrolase (BSH). Primary BA such as CDCA can be converted to secondary BA such as UDCA by microbial 7α-hydroxysteroid dehydrogenase (7α-HSDH) and also to LCA by 7α-dehydrogenase. The Clostridium family readily converts CDCA to UDCA due to the expression of 7α-HSDH (Wei et al., 2020). *Lactobacillus plantarum* strain H-87 exhibits a good ability to hydrolyze glycosyl glycochenodeoxycholic acid (GCDCA) and increase CDCA concentration in the ileum, and thus inhibit fat accumulation and insulin resistance. *Lactobacillus yohimbe* as a BSH-producing bacterium can alter the level of BSH to balance the composition of different BA members and thus regulate FXR activity(Yang and Wu, 2022). Conversely, BA likewise affect the composition of the host gut microbiota. For example, CA administration to normal diet-fed mice resulted in a gut microbiota similar to that of obese mice (Jia et al., 2021).

### Receptors of secondary bile acids

The receptors for secondary bile acids include the farnesoid X receptor (FXR), G protein-coupled receptor (GPCR), and TPH (TGR5; GPBAR1; Zwartjes et al., 2021). FXR is expressed in a variety of cells, including hepatocytes, adipocytes, enterocytes, and pancreatic islet cells, and is activated by CDCA and DCA (Zwartjes et al., 2021). FXR is essential for lipid metabolism, especially in the liver (Sinal et al., 2000; Watanabe et al., 2004). Targeting FXR has beneficial effects on body weight, glucose homeostasis, energy expenditure, and inflammation. Another bile acid receptor, GPBAR-1, is widely expressed in the human body, such as in the gastrointestinal tract, adipose tissue, liver, and gallbladder. Similarly, it can also be activated by taurocholic acid. GPBAR-1 is involved in glucose metabolism, energy metabolism, and inflammatory processes in the body and may play an indirect role in lipid metabolism by regulating lipid absorption in the intestinal lumen (Portincasa et al., 2020).

### Secondary bile acids promote brown fat activation to improve metabolism

In white and brown adipose tissues, bile acids induce the transcription of cAMP-PKA-CREB-induced Dio2 through the TGR5 receptor. Dio2 then promotes 2-iodothyronine deiodinase (D2) production, which triggers the conversion of inactive thyroxine (T4) to T3 (the active form of thyroid hormone), thereby increasing thermogenesis in the thermogenic tissue (Watanabe et al., 2006). FEX (FXR activators) can induce LCA production by intestinal flora (for example, Acetatifactor and Bacteroidetes), activate TGR5, and stimulate GLP-1 secretion in intestinal L cells (Pathak et al., 2018). Intestinal hypoxia-inducible factor 2α (HIF-2α) leads to a decrease in intestinal lactate levels by controlling the expression of intestinal LDHA genes (Wu et al., 2021); this also led to a decrease in vulva-like bacilli and an increase in the abundance of rumen cocci. These changes lead to elevated levels of taurine-conjugated bile acid (TCA) and DCA, resulting in the activation of TGR5. This activation upregulates the expression of coupling protein (UCP) 1 and mitochondrial creatine kinase (CKMT) 2, subsequently inducing thermogenesis in WAT (Wu et al., 2021). Altering the BA pool by increasing microbes with 7α-dehydroxylation in gut microbes improved the phenotype of obese mice by increasing non-12OH-BAs(TCDCA, TUDCA) and decreasing 12OH-BAs(TCA). It was shown that taurochenodeoxycholic acid (TCDCA) induced the expression of UCP1, PGC1α, and Dio2 in WAT and BAT, while TCA had no significant effect on energy metabolism in adipose tissue (Dos Reis Araujo et al., 2022). Non-12OH BA improved the energy metabolism of white and brown fat through TGR5-mediated activation of BAT and upregulation of UCP1 expression. Oral administration of CDCA to healthy subjects also increased energy expenditure by activating TGR5 in brown adipocytes. Therefore, manipulation of TGR5 in adipocytes may be considered as a promising target for the alleviation of obesity.

## Tryptophan metabolites

Tryptophan is an essential aromatic amino acid with the largest relative molecular weight of the 20 common canonical amino acids. Tryptophan is a biosynthetic precursor of a large number of microbial and host metabolites (Alkhalaf and Ryan, 2015). It is primarily obtained from foods, such as peanuts, millet, walnuts, black beans, milk, bananas, cheese, bread, poultry, and black sesame (Agus et al., 2018). To date, there have been no reports of adverse effects of excess tryptophan in the diet.

### Tryptophan metabolic pathway

Nearly 95% of tryptophan is degraded to kynurenine (KYN), kynurenic acid (KNA), and quinolinic acid (QA) *via* the kynurenine pathway, which plays a key role in immune regulation, inflammatory responses, and neural signaling (Clarke et al., 2013). This process is regulated by two rate-limiting enzymes: tryptophan 2,3-dioxygenase (TDO) in the liver and indoleamine 2,3-dioxygenase (IDO) in extrahepatic tissues (Cervenka et al., 2017). Several intestinal bacteria encode enzymes that are homologous to those of the eukaryotic Kyn pathway, and these intestinal bacteria include Pseudomonas, Xanthomonas, Burkholderia, and other species (Vujkovic-Cvijin et al., 2013). About 4%–6% of tryptophan is directly converted by gut microbes into molecules, such as indole and its derivatives. Indole-3-aldehyde (IALD), indole-3-acid-acetic acid (IAA), indole-3-propionic acid (IPA), indole-3-acetaldehyde (IAAld), and indoleacrylic acid are ligands for the aryl hydrocarbon receptor (AHR; Neavin et al., 2018). AHR, a cytoplasmic ligand-activated transcription factor, is a key regulator of immunity and inflammation, maintaining intestinal homeostasis by affecting adaptive immunity and mucosal barrier function (Shinde and McGaha, 2018). It also acts as a ligand for endogenous tryptophan metabolites, such as KYN and cinnabarinic acid (CA; Neavin et al., 2018; Shinde and McGaha, 2018). Exogenous substances, such as food, activate AHR signaling, which acts on epithelial renewal, barrier integrity, and immune cells [Th17 cells, intrinsic lymphocytes, macrophages, dendritic cells (DCs), and neutrophils] to maintain intestinal homeostasis (Lamas et al., 2018). Different bacterial species have different tryptophan catalytic enzymes. Bacteria such as *Bacteroides ovatus*, *Clostridium limosum*, *Enterococcus faecalis,* and *Escherichia coli* convert Trp to indole. The oxidative and reductive pathways in *Clostridium sporogenes* lead to the production of IAA and IPA. *Peptostreptococcus* spp. Converts Trp to IA and IPA. *Ruminococcus gnavus* converts Trp to tryptamine *via* Trp decarboxylase. *Clostridium sporogenes*, which decarboxylates Trp to the neurotransmitter tryptamine (Su et al., 2022).

About 1%–2% of ingested tryptophan is converted to 5-hydroxytryptamine (5-HT)/serotonin by the 5-HT pathway. 5-HT, produced in the gut and brain, is a multifunctional indolamine whose biosynthesis has been conserved (Yabut et al., 2019). 5-HT is synthesized from L-tryptophan by a two-step reaction involving 5-HTP (TPH1) and aromatic amino acid decarboxylase (Gao et al., 2018). It takes part in the regulation of energy balance, appetite, gut motility, immunity, liver repair (Yabut et al., 2019), and cardiovascular and pulmonary physiology in the human body (Berger et al., 2009). Here, we focus on peripheral 5-HT, which is unable to cross the blood–brain barrier under physiological conditions (Yabut et al., 2019). Most peripheral 5-HT is produced by enterochromaffin cells (Ecs) in the intestine and stored in platelets (Figure 1; Jones et al., 2020). Studies have demonstrated that fasting and feeding high-carbohydrate or high-fat foods to mice increases plasma 5-HT levels (Stephen and Abraham, 2019). Peripheral 5-HT acts as a signaling molecule in the gastrointestinal tract to activate the corresponding receptors and participate in a variety of human physiological activities. There are over 14 types of 5-HT receptors (HTR), all of which are G protein-coupled receptors, such as 5-hydroxytryptamine receptors 2A and 2 B (HTR2A; HTR2B), except for HTR3, which is a ligand-gated cation channel. New evidence also points to a key role of the gut microbiota in regulating host 5-HT secretion in EC cells by interacting to various compounds produced from the host or gut microbes. Injection of SCFAs into the proximal colon significantly increased the release and production of 5-HT. SCFAs also enhanced colonic TPH1 expression by acting on EC cells (Liu et al., 2021).

### Effects of tryptophan on adipose tissue

**Figure 2:**
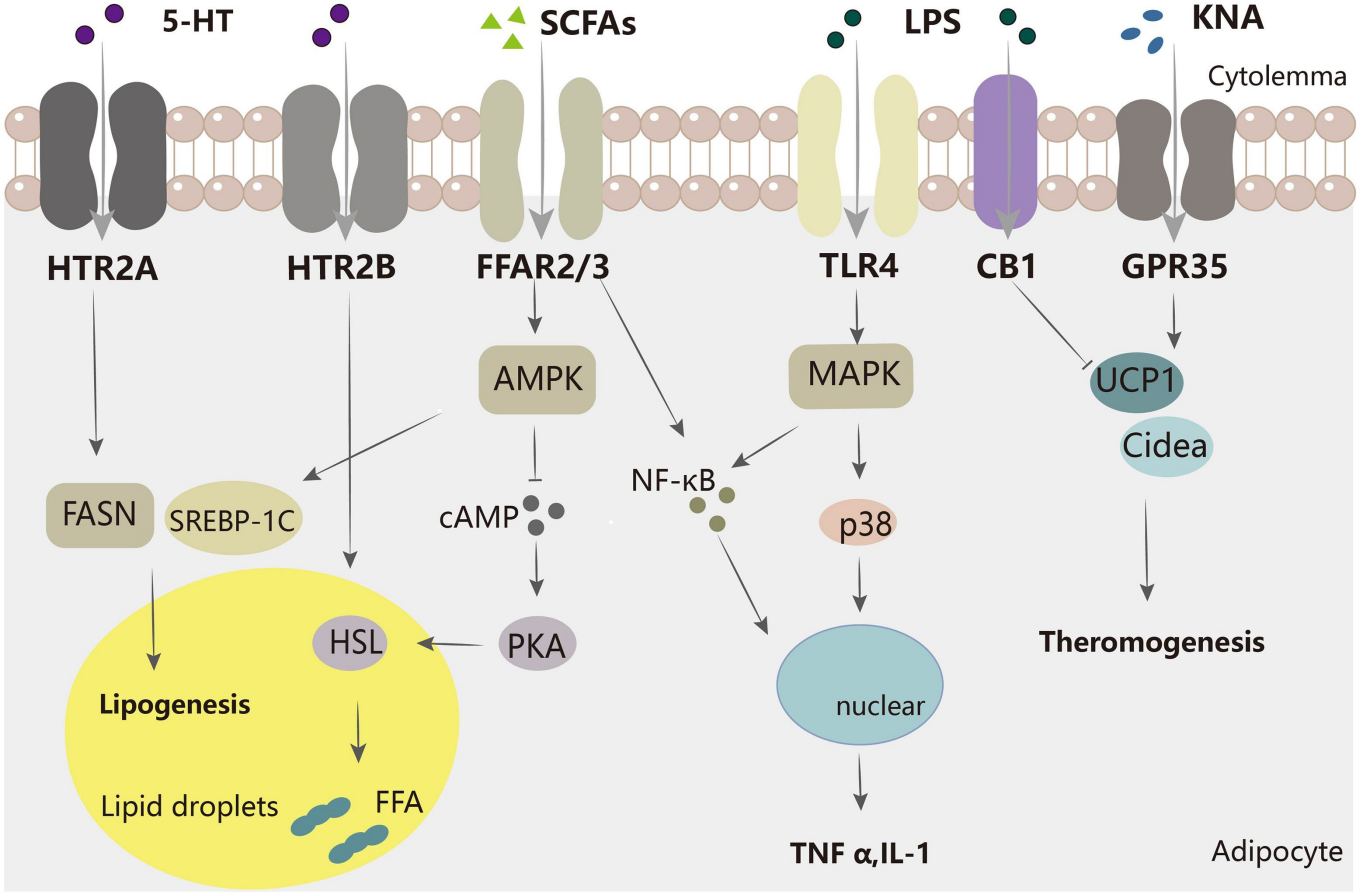
Mechanisms of several major intestinal microflora metabolites regulating adipocytes. SCFA inhibits the AMPK-cAMP-PKA pathway *via* FFAR2/3, decreasing HSL and thereby inhibiting lipolysis. SCFA also increases adipogenesis-related genes, such as SREBP-1C.5-HT acts on FASN protein through HTR2A to increase adipogenesis, and through HTR2B to promote HSL activation to increase lipolysis. LPS acts on TLR4 and activates the MAPK pathway to promote inflammation, and SCFA can also facilitate inflammation by increasing inflammatory molecules such as NF-κB. KNA promotes the expression of thermogenesis-related genes, while LPS has the opposite effect.

KNA can serve as an early biomarker for diabetes (Favennec et al., 2015) and is significantly elevated in the obese population (Nam et al., 2019; Pyun et al., 2021). It was inferred that there is only a positive link between high KNA and BMI or the insulin resistance index HOMA2-IR in overweight individuals. In addition, their adipose tissue had elevated gene expression levels of IDO1 and its downstream enzymes, such as KYN, kynurenine aminotransferase (KAT), and kynurenine 3-monooxygenase (KMO; Favennec et al., 2015). However, in another study, serum KNA levels were negatively correlated with BMI (Pyun et al., 2021). These contradicting observations may be due to different criteria, with the former using multivariable linear regression models and the latter using Pearson’s coefficient on an online calculator. In addition, Agudelo et al. demonstrated that KNA increases cellular respiration and β-adrenergic signaling through GPR35 (Figure 2), stimulating the expression of genes involved in adipose metabolism and thermogenesis in adipose tissue (Agudelo et al., 2018). KNA also plays an anti-inflammatory role by activating GPR35 and inducing autophagy-dependent degradation of NLRP3 in macrophages (Barth et al., 2009; Zheng et al., 2019). These studies suggest that the kynurenine metabolic pathway has the potential to be a new therapeutic target for the control of energy homeostasis.

Whole blood 5-HT is significantly negatively correlated with BMI, waist circumference, waist-to-hip ratio, and total % body fat (Hodge et al., 2012); this finding suggests that 5-HT may be useful in improving metabolic syndrome. Gut-derived 5-HT induces satiety in concert with peripheral signals, such as cholecystokinin and leptin (Voigt and Fink, 2015). In mice, gut-derived 5-HT is upregulated during fasting and facilitates lipolysis and gluconeogenesis in the liver. As shown in Figure 2, gut-derived 5-HT signals through the HTR2B receptor and promotes lipolysis by increasing the phosphorylation and activation of hormone-sensitive lipases (Sumara et al., 2012). This suggests that we can improve type 2 diabetes by inhibiting 5-HT synthesis. Another study showed that TPH1 gene-deficient mice fed a high-fat diet defended against obesity, insulin resistance, and non-alcoholic fatty liver disease (NAFLD) through a mechanism that involved greater energy expenditure in thermogenic brown adipose tissue. 5-HT weakened beta-adrenergic induction of thermogenic programs in brown and beige adipocytes *in vitro*. Under HFD, inhibition of 5-HT synthesis reduced weight gain, improved glucose tolerance, increased thermogenic activity in BAT, and decreased adipogenesis in WAT. 5-HT inhibits BAT thermogenesis *via* HTR3 and increases WAT lipogenesis *via* HTR2A (Oh et al., 2015). 5-HT also increases *de novo* adipogenesis in adipose tissue. Compared to low-5-HT rats, the protein level of the major adipose synthase FASN was increased in the adipose tissue of high-5-HT rats, which suggests increased adipogenesis in WAT of high-5-HT animals (Figure 2). Additionally, adipose tissue-specific knockout of Tph1 or Htr2a (Tph1 FKO) mice fed an HFD exhibited reduced lipid accumulation, increased thermogenesis, and resistance to obesity. This also implies that the effect of 5-HT on adipogenesis is mediated by its receptor, HTR2A (Shong et al., 2020). However, MRNA levels of genes responsible for adipocyte differentiation did not differ significantly between the 5-HT sublines (Nonogaki, 2022). In summary, tryptophan metabolism in the gut plays an important role in governing energy homeostasis and may be a therapeutic target for obesity and metabolic diseases. However, further studies on humans are still needed because of the complexity of microbial-host interactions. The utilization of tryptophan-metabolizing microbes or inhibition of 5-HT signaling in adipose tissue may be an effective therapeutic approach for obesity.

## Trimethylamine-N-oxide

### Sources of TMAO

Nutrients present in high-fat foods (phosphatidylcholine, choline, L-carnitine, and γ-butyrobetaine) are first metabolized by intestinal microbial enzymes, such as CutC/D21, 22CntA/B23, and YeaW/X15, to produce the major bacterial metabolite, trimethylamine (TMA; Schugar et al., 2018). Trimethylamine-N-oxide (TMA) is further metabolized in the liver by the host enzyme flavin monooxygenase 3 (FMO3) to trimethylamine-N-oxide (TMAO; Figure 1). The TMAO pathway has previously been identified as a potential influential element in the pathogenesis of obesity, CVD, alcohol-associated hepatitis, and type 2 diabetes (Schugar and Brown, 2015; Petriello et al., 2016; Shih et al., 2019; James et al., 2022; Song et al., 2022). TMAO was related to the fecal microbiome, and the genera *Butyribrio*, *Roseburia*, *Coprobacillus*, and *Catenibacterium* were enriched in individuals in the lowest versus highest TMAO tertile (James et al., 2022).

### Effect of TMAO on adipose tissue

Studies have shown that TMAO is related to energy metabolism in the adipose tissue. Rebecca et al. observed that elevated systemic levels of TMAO were associated with type 2 diabetes in humans. Plasma TMAO levels in mice and TMAO-producing enzyme FMO3 mRNA expression in men were positively correlated with obesity. FMO3 protected mice from HFD-induced obesity by stimulating the browning of white adipose tissue (Schugar et al., 2017). Furthermore, gut microbial TMAO is associated with insulin signaling. Both TMA and TMAO treatment resulted in the altered phosphorylation of protein kinase A (PKA) signaling-related proteins, including A-kinase anchor protein 12 (AKAP12) and the regulatory subunit of PKA (Prkar1a). Moreover, TMA and TMAO promote the phosphorylation of insulin-like growth factor 2 (IGFR2; Schugar et al., 2018). TMAO induced hyperphosphorylation of synaptosome-associated protein 23 (Snap23), which promoted insulin-stimulated glucose transporter protein transport to the cell surface in adipocytes (Grusovin and Macaulay, 2003; Li et al., 2013).

## Lipopolysaccharides

Lipopolysaccharides (LPS), also known as bacterial endotoxins, are major components of the outer wall layer of gram-negative bacteria and vary across intestinal strains. LPS generally consists of an O-polysaccharide chain, R-nucleus, and lipid-like A fraction. The main lipid-like A fraction mediates the toxic and immunomodulatory properties of lipopolysaccharides (Santos et al., 2003; Kaszowska et al., 2017). Different intestinal flora produce different endotoxin structures, and a number of them cause inflammatory responses. However, *Bacteroides* endotoxins seem to suppress immune stimulation and protect cells against *E. coli* endotoxin-induced inflammatory responses (Vatanen et al., 2016). LPS is transported from circulation to the surrounding adipose tissue by binding to lipoproteins. LPS-binding protein (LBP) catalyzes the transfer of endotoxin to lipoproteins, which transfers endotoxin from endotoxin micelles to soluble CD14 (sCD14) in plasma and from the endotoxin-sCD14 complex to HDL and other lipoproteins (Wurfel and Wright, 1997).

### LPS receptor signaling

Toll-like receptors (TLR4) are LPS receptors expressed in all somatic cells. TLRs are an important class of protein molecules involved in natural immunity, triggering host immune responses, and enhancing adaptive immunity (Hornung et al., 2002; Fitzgerald and Kagan, 2020). LPS activates two pathways through TLR4: the myeloid differentiation factor 88 (MyD88)-dependent pathway (which activates NF-κB and promotes the secretion of pro-inflammatory cytokines) and the MyD88 non-dependent pathway. Lipopolysaccharides can also activate TLR2 in adipocytes, leading to the upregulation of GPR109a (especially in macrophages; Warner and Núñez, 2013).

### Effect of LPS on adipose tissue

LPS regulates local adipose tissue inflammation (Figure 2). Circulating endotoxins cause endotoxemia and trigger chronic low-grade inflammation (Cani et al., 2008) such as *E. coli*-derived LPS. However, metabolically beneficial endotoxemia promoted by *R. sphaeroides* LPS counteracts the glucose abnormalities induced by equal doses of *E. coli* LPS and improves insulin sensitivity in obese mice. *R. sphaeroides* LPS increased insulin-stimulated AKTser473 phosphorylation in adipose tissue, improved insulin signaling, and alleviated dysglycemia in obese mouse adipose tissue (Anhê et al., 2021).In wild-type (WT) mice, acute intravenous administration of endotoxin in subcutaneous fat depots significantly increased the mRNA expression of IL-6, PAI-1, and IL-1. This effect may be triggered by its binding to a complex of mCD14 and TLR4 on the surface of natural immune cells (Luche et al., 2013). CD14, a key molecule in innate immunity, is expressed abundantly on the surface (mCD14) of mature monocytes, macrophages, and neutrophils (Kitchens and Thompson, 2005). Luche et al. also showed that metabolic endotoxemia promotes the proliferation of pre-adipocytes and the local infiltration of macrophages (including increased numbers and conversion to M1 type) through a CD14-dependent mechanism. Unno et al. found that an increased concentration of *Acinetobacter baumannii*-derived LPS enhanced the expression of several adipokines (e.g., MIP-2, MCP-1, TNF-α, IL-6, lipocalin-2, and FABP4) and significantly decreased the expression of leptin and lipocalin in 3 T3-L1 adipocytes. As hypertrophic adipocytes die and are replaced by inflammatory cells, an existing study showed that endotoxin activates caspase-4/5/11 and induces a TLR4-dependent immune response, leading to NLRP3 inflammatory vesicle-dependent caspase-1 activation and obese cell death (Giordano et al., 2013). In addition, LPS plays a negative role in the control of adipogenesis and inflammation through the endogenous cannabinoid system (ECBS). ECB is widely expressed *in vivo*, including in the pancreas, muscle, gut, adipose tissue, liver, and hypothalamus (Cani et al., 2014). Two cannabinoid receptors are known: CB1 (present in the brain, adipose tissue, and skeletal muscle) and CB2 (present in immune cells; Alexander et al., 2019). Treatment of ob/ob mice with a specific antagonist (SR141716A) to block the CB1 receptor developed impaired intestinal permeability and metabolic endotoxemia. Lipolytic dysregulation is more likely to occur during endotoxemia and inhibit white adipose tissue browning (Okla et al., 2015). The above results reveal that reducing gut flora production by LPS may be an entry point for obesity mitigation.

## Conclusion and perspective

The roles of gut microbes and their derivatives in metabolic diseases have been extensively investigated and validated in a growing number of models and clinical trials. In this review, we focused on the available literature on the regulation of metabolic processes, including lipolysis, adipogenesis, browning, and adipose tissue dysfunction, in adipose tissue by gut flora metabolites (Table 1). Many complex mechanisms underlying the role of the gut microbiota in regulating host metabolism and energy homeostasis have been identified to date, providing a solid foundation and direction for subsequent studies on metabolic diseases.

In recent years, research has focused on the role of nutrients, drugs, and probiotic interventions related to the gut flora to overcome obesity and other metabolic disorders. However, studies regarding their application as alternatives to drugs in clinical treatment are ongoing. Critically, there are conflicting results regarding the available research data. There are species differences between humans and rodents, including anatomical, physiological, pathophysiological, and gut flora. Interindividual differences in flora between humans also need to be considered in clinical trials. Therefore, long-term human translational studies and clinical trials are needed in the future to help determine the most appropriate method for administration, dose, duration, as well as other factors. To better exploit the biological mechanism by which microbial metabolites affect adipose tissue and other organs, researchers will need to resort to more advanced techniques and tools, such as metabolomic analysis of serum and feces from clinical subjects and genomic analysis of new strain types.

## Author contributions

XJ and YX: conceptualization. WYL, PYL, and GY: methodology. WYL and GY: software. WYL and GY: writing–original draft preparation. WYL and PYL: data curation. PYL, YX, and XJ: writing–review and editing. YX and XJ: funding acquisition. All authors read and approved the manuscript.

## Funding

This work was supported by the National Natural Science Foundation of China (grant number 82170369), Jilin Provincial Science and Technology Foundations (20210509003RQ), and the program of Changchun Science and Technology Bureau Development Plan project (grant number 21ZY29).

## Conflict of interest

The authors declare that the research was conducted in the absence of any commercial or financial relationships that could be construed as a potential conflict of interest.

## Publisher’s note

All claims expressed in this article are solely those of the authors and do not necessarily represent those of their affiliated organizations, or those of the publisher, the editors and the reviewers. Any product that may be evaluated in this article, or claim that may be made by its manufacturer, is not guaranteed or endorsed by the publisher.
